# Feasibility, effectiveness and costs of a decision support intervention for consultees and legal representatives of adults lacking capacity to consent (CONSULT): protocol for a randomised Study Within a Trial

**DOI:** 10.1186/s13063-022-06887-5

**Published:** 2022-11-24

**Authors:** Victoria Shepherd, Fiona Wood, Katie Gillies, Adam Martin, Abby O’Connell, Kerenza Hood

**Affiliations:** 1 Centre for Trials Research, Cardiff University, Cardiff, UK; 2 PRIME Centre Wales, Wales, UK; 3 Division of Population Medicine, Cardiff University, Cardiff, UK; 4 Health Services Research Unit, University of Aberdeen, Aberdeen, UK; 5 Academic Unit of Health Economics, University of Leeds, Leeds, UK; 6 Exeter Clinical Trials Unit, University of Exeter, Exeter, UK

**Keywords:** Informed consent, Clinical trial, Proxy, Decision-making, Study Within A Trial (SWAT)

## Abstract

**Background:**

Randomised trials play a vital role in underpinning evidence-based care. However, trials involving adults with impaired capacity to consent raise a number of ethical and methodological challenges, leading to the frequent exclusion of this group from trials. This includes challenges around involving family members as alternative ‘proxy’ decision-makers. Family members are often given little information about their role as a consultee or legal representative. Some family members find making a decision about trial participation difficult and may experience an emotional and decisional burden as a result. Families have reported a need for greater support and guidance when making such decisions, leading to the development of a decision aid (‘Making decisions about research for others’) for family members acting as consultee/legal representative. The decision aid now requires evaluation to determine its effectiveness in supporting families to make more informed decisions.

**Methods:**

This protocol describes a prospective, multi-centre, randomised-controlled Study Within a Trial (SWAT) to evaluate the effectiveness of the decision aid. The SWAT will initially be embedded in approximately five host trials. SWAT participants will be randomised in a 1:1 ratio to either the intervention (decision aid alongside standard information about the host trial provided to consultees/legal representatives) or control (standard information alone). The primary outcome is the quality of proxy consent decision, assessed by the Combined Scale for Proxy Informed Consent Decisions (CONCORD). The SWAT design is informed by previous qualitative research. Initial feasibility will be explored in one host trial, followed by the main SWAT. An embedded process evaluation and economic evaluation will enable the SWAT findings to be contextualised and identify factors likely to affect implementation.

**Discussion:**

This SWAT will generate the first evidence for recruitment interventions for trials involving adults lacking capacity to consent and add to knowledge about the use of decision support interventions in trial participation decisions. The SWAT will be embedded in a range of trials, and the heterogenous nature of the host trials, settings and populations involved will enable the intervention to be evaluated in a wide range of contexts. However, a pragmatic and flexible approach to conducting the SWAT is needed.

**Trial registration:**

The SWAT is registered as SWAT #159 with the Northern Ireland Hub for Trials Methodology Research SWAT repository (registered 09.08.2020). Each host trial will be registered on a clinical trials registry.

## **Background**

Promoting inclusivity in research is at the heart of national research strategies [1], such as the UK Government’s ‘Future of UK Clinical Research Delivery’ [2]. The under-representation of groups such as people who lack decision-making capacity has been recognised internationally as a concern, including by the UK’s National Institute for Health and Care Research (NIHR) who are developing innovations in clinical trial design and delivery to increase recruitment of those groups [3], and by a drive for more inclusive research in the USA [4]. Identifying the best approaches to ensure the inclusion and participation of under-represented or vulnerable groups in randomised trials is a recognised priority area [5].

An estimated two million people in England and Wales have significantly impaired decision-making through dementia, learning disabilities, or other conditions affecting cognitive function such as delirium, multimorbidity, or critical illness [6]. Research into conditions affecting these groups who often experience higher care needs is vital; however, adults who lack capacity to consent are often excluded from research [7–9]. Despite a growing emphasis on making research more inclusive to under-represented or underserved populations [3], few trials are designed to include participants who lack capacity, and the numbers of participants unable to consent who are actually recruited by trials designed to include this population are worryingly low [10].

Trials involving adults who lack capacity present a number of ethical, legal, and practical challenges [11–14]. Reported challenges include the complexity of the legal frameworks governing research involving adults who lack capacity to consent and the provisions for an alternative ‘proxy’ decision-maker to be involved in decisions about their participation [12]. Legal arrangements for the involvement of proxies differ between jurisdictions and according to the type of research, which creates complexities when conducting national and international trials involving adults lacking capacity [15, 16]. For UK clinical trials of medicinal products, this is a legal representative, usually a family member or friend, who provides informed consent based on the person’s ‘presumed will’ [17]. For other types of research, under the Mental Capacity Act 2005 in England and Wales, a family member or friend acts as a consultee and provides advice to the researchers about what the person’s wishes and feelings would be about taking part [18]. However, partly due to this complexity, researchers and healthcare professionals have difficulty interpreting the legal requirements [16]. This results in poor communication with proxies about what their role is in trial participation decisions [19].

### Challenges of making proxy decisions about trial participation

Other challenges arise from the psychological stress and uncertainty that family members may experience when asked to take on this role [20, 21]. Previous research has shown that family members acting as consultees or legal representatives express uncertainty about making what many can find to be complex and challenging decisions which can result in decisional and emotional burden [22, 23]. Proxy decision-making for research has been demonstrated to be stressful in some settings [24], and some studies have reported that nearly all proxies experience some degree of burden when making decisions about research [25]. This leads to a high proportion of families declining participation [26]. Despite numerous innovations to improve informed consent processes for research, there are no interventions for proxies who are making decisions about non-emergency research on behalf of someone who lacks capacity.

### Development of a decision support intervention for proxy decision-makers

Decision support interventions, also known as decision aids (DAs), are increasingly used to support patients making decisions about healthcare treatment or uptake of screening [27]. More recently, DAs have been developed for people considering participating in clinical trials [28]. DAs support the decision process by providing structured guidance on steps of decision-making, information about available options and their associated outcomes, and information that enables patients to consider what value they place on particular outcomes [29]. A novel DA for proxy decision-making about research has been developed in collaboration with lay advisors and stakeholder groups and informed by theoretical frameworks and empirical research [30]. The DA has undergone acceptability testing with both those who would deliver and receive the intervention. It now requires evaluating to assess whether it provides an effective form of support to families making non-emergency trial participation decisions.

### Evaluating the decision support intervention in ‘Study Within A Trial’

The CONSULT study will evaluate proxy decision-making by families of adults who lack capacity to consent when provided with the DA alongside standard study information, compared to standard study information alone. It will be evaluated as a ‘Study Within a Trial’ (SWAT). A SWAT is a self-contained research study embedded within a host trial with the aim of evaluating alternative ways of delivering or organising a particular trial process, typically recruitment or retention strategies [31]. SWATs can be conducted across multiple host trials, either at the same time or sequentially. Whilst ideally they are built into the host trial from the start, a SWAT can be included in an ongoing trial and need not run for the whole duration of the host trial [31]. This approach, which was recently used in the Medical Research Council Systematic Techniques for Assisting Recruitment to Trials (MRC START) programme, enables a more precise estimate of the effect of the strategy through meta-analysis across multiple trials and exploration of the degree to which the effects of recruitment strategies varies across different trial contexts [32].

As this is the first intervention for families making research participation decisions on behalf of adults who lack capacity, and the first SWAT involving proxy decision-makers rather than participants themselves, a feasibility stage will first be conducted in accordance with MRC guidelines for complex interventions [33]. The feasibility stage will inform the main SWAT through testing the feasibility of the intervention (provision of the DA alongside standard information about the host trial provided to consultees/legal representatives), the proposed outcome measures, and of conducting a SWAT to evaluate the intervention. As it progresses to the main SWAT, an embedded process evaluation will address issues regarding reach, contamination, context, adaptation and fidelity to the intervention [34] and an economic evaluation will also be embedded to identify and measure the resources involved in delivering the intervention [35]. If effective, the DA would support families of adults who lack capacity to consent to make more informed decisions about participation, and reduce the decisional burden they experience through addressing uncertainty [36], and so support greater inclusiveness in research.

### Establishing the acceptability and feasibility of conducting the ‘Study Within A Trial’

We have previously described the ethical and methodological considerations we encountered when designing this SWAT for trials involving adults lacking capacity [37] and studies exploring family members’ views about the acceptability and feasibility of the DA (DECISION and DECISION 2) [30, 38]. To inform the development of the CONSULT SWAT, we conducted a qualitative study (CONSULT-ENABLE) to explore researchers’ and healthcare professionals’ views about the acceptability and feasibility of conducting the SWAT, and how the DA might be implemented in practice. The study also explored the barriers and facilitators to conducting trials involving adults lacking capacity, the design of the qualitative study and findings relating to this aspect have been previously reported [12]. Interviews were conducted with 26 UK researchers and healthcare professionals with experience in a range of roles, trial populations and settings. The interviews identified a number of key findings that are summarised below and incorporated into the SWAT design (see Table 1).

**Table 1:** CONSULT-ENABLE key findings to inform SWAT design (expanded from Shepherd et al 2022 [12])

**SWAT design component and key findings**	
**Selection of host trials**	
Whilst the DA was considered applicable to all study types, it was thought to be particularly useful for more complex and burdensome studies that have a higher decisional burden for consultees, and less suitable for acute or emergency trials with short recruitment windows.	
**Consent process**	
Researchers stressed the need to reduce the informational and consent burden for SWAT participants by avoiding a ‘double consent process’. Requiring an extra layer of consent for the SWAT might deter people from participating in the SWAT and acting as a consultee/legal representative host trial. Given the low-risk nature of the study and the impracticability of gaining consent prospectively prior to randomisation or receiving the intervention, consent to providing data was considered appropriate. This could be achieved through the return of the questionnaire indicating consent to participate. Researchers stressed the importance of harmonising information provided to family members about the host trial and SWAT in order to create a ‘whole package’ and improve clarity.	
**Level of randomisation**	
In addition to the usual issues relating to decisions about the appropriate level of randomisation (i.e. cluster or individual), specific factors to consider in trials involving participants with impaired capacity include that more than one researcher may be involved in seeking consent or consultee involvement, and in settings such as care homes there may be one or multiple researchers recruiting at each care home. Therefore, particular attention to allocation processes is needed in order to reduce the burden for host trials and minimise the risk of contamination.	
**Intervention delivery**	
The method of delivery of the DA needs to be aligned with the host trial recruitment process as, depending on the context, trials may approach consultees/legal representatives in person or by phone or may post out information with families sometimes having the option to speak to a researcher or just return the consent/declaration form by post with no contact with researchers.	
**Data collection**	
As with other study processes described previously, data collection processes need to minimise the additional burden for researchers and participants involved. This must be balanced with the need to collect information not normally collected by the host trial such as personal information from a consultee.	

## Methods

This SWAT protocol is written in accordance with the guidelines for reporting embedded recruitment trials outlined by the MRC START Group [39]. The protocol has been pre-registered on the Northern Ireland Hub for Trials Methodology Research SWAT repository (Queen’s University Belfast) (SWAT ID #159) [40]. The overarching SWAT has received ethical approval (Leeds West REC ref. 22/YH/0121). Each host trial will obtain approval for embedding the SWAT, either from the start of the trial or as a substantial amendment after the host trial has commenced.

### Study design

**Fig. 1:**
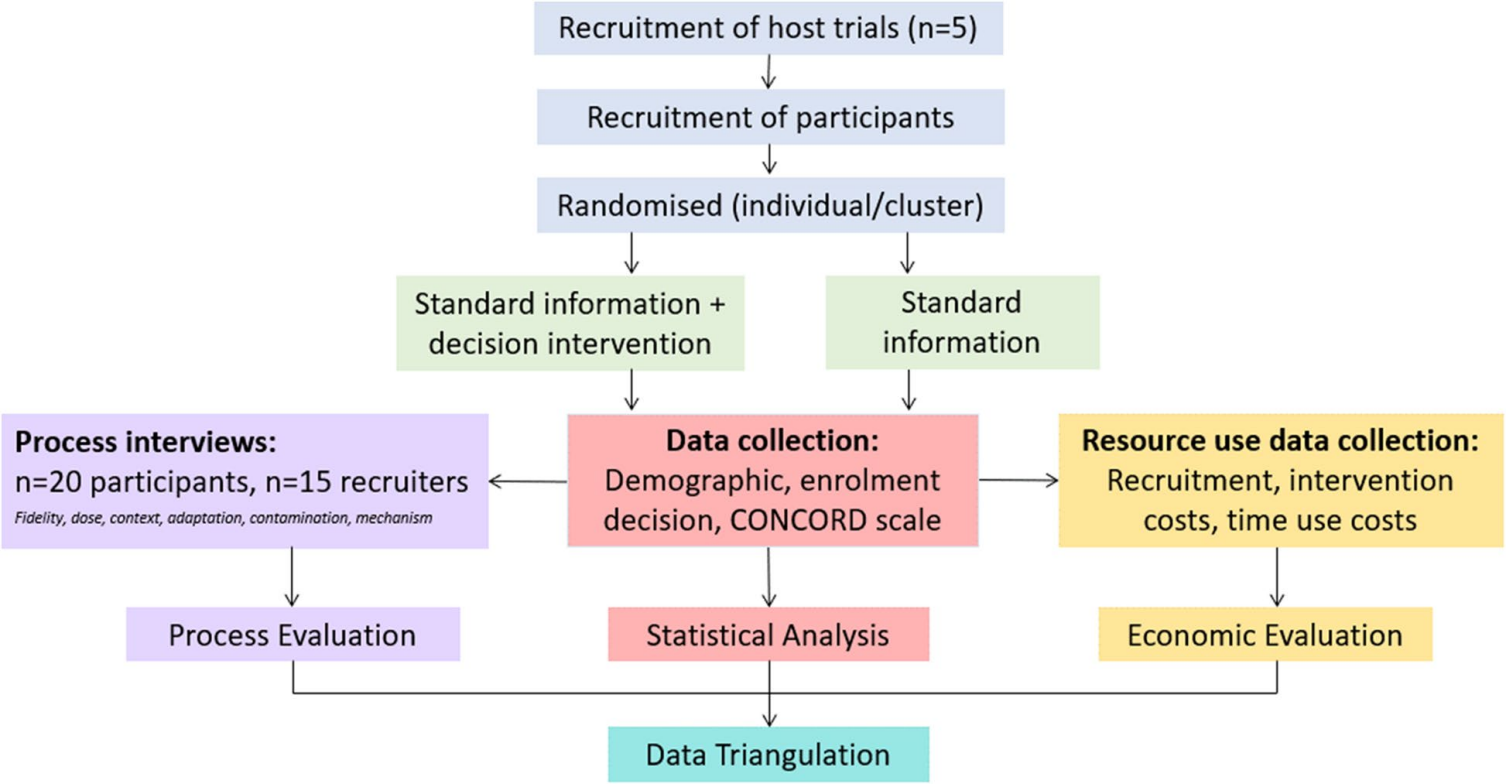
CONSULT SWAT trial design

The CONSULT SWAT is a two-arm, parallel-group, embedded randomised-controlled trial to investigate the effect of a decision support intervention compared with standard study information on decision-making by consultees and legal representatives of adults lacking capacity to consent. The CONSULT study uses established SWAT methodology [31] to evaluate the intervention in approximately 5 host trials that recruit adults who lack capacity through personal consultee or personal legal representative involvement (see Fig. 1).

An initial feasibility stage will be conducted in one host trial to test the intervention and SWAT with 20 family members and up to 15 research staff (recruiters and members of the trial co-ordination team). This is an internal randomised pilot study [41] which will explore the feasibility of SWAT procedures including outcome measures, establish acceptability and identify any unintended consequences, help estimate the likely rates of recruitment and retention of participants for the SWAT, and give an understanding about likely SWAT sample sizes. There are generally no formal a priori sample size calculation for SWATs [31]. The SWAT will be undertaken on the basis of the maximum number of recruiters and participants possible per host trial. The number of host trials anticipated (approx. n=5) has been informed by previous co-ordinated programmes that have simultaneously embedded a SWAT in multiple host trials (e.g. [32, 42–44]).

An embedded process evaluation and economic evaluation in the SWAT will enable the findings to be contextualised in order to draw robust conclusions about the effectiveness of the intervention and factors likely to affect implementation. Process evaluations enable an understanding of the causal assumptions underpinning an intervention and are a vital part of the use of evaluation to understand how interventions work in practice [45]. In addition to quantitative data such as data completion rates, approximately 20–25 participants from the intervention arm and 15 recruiters will be interviewed. The interviews will be conducted across host trials and will provide more in-depth information about how the intervention works in different trial contexts. The economic evaluation will take the form of a cost-consequence analysis (CCA) where disaggregated costs and a range of outcomes are presented [35]. The CCA will take an NHS and societal perspective by identifying and measuring the resources involved in intervention production and delivery as well as resource use, including time use, by family members and research staff.

### Objectives

The objectives for the feasibility stage of the SWAT are to:

- Establish the feasibility of the novel decision support intervention in a real (rather than hypothetical) decision-making situation
- Establish the feasibility of conducting a SWAT
- Establish the feasibility and acceptability of the CONCORD scale in a real (rather than hypothetical) decision-making situation and of measuring resource use

The feasibility stage will inform the main SWAT and help to determine whether any changes to SWAT processes are required, rather than being assessed against formal ‘stop/go’ criteria.

The objectives of the main SWAT are to:

- Evaluate the effectiveness of the decision support intervention through a randomised SWAT
- Explore issues affecting future implementation such as reach, contamination, context, adaptation and fidelity through an embedded process evaluation
- Undertake an economic evaluation through a CCA to explore the resources involved in intervention delivery

### Eligibility criteria

Host trials can be based in any setting, including in primary or secondary care, the community, or care homes. Host trials will be eligible if the trial team anticipates that a reasonable proportion of potential participants will lack capacity to consent and will involve personal consultees or personal legal representatives. Trials will be ineligible if they only involve recruitment without prior consent (‘deferred’ consent), use only nominated consultees or professional legal representatives, or if the participation decision needs to be made urgently or within a short timeframe (i.e. emergency research). In order to provide a greater understanding about the factors that influence the effectiveness and implementation of the intervention, host trials and sites may be purposively selected to participate in the SWAT where ongoing analysis of data from the SWAT or qualitative interviews suggests that conducting the SWAT in specific trial contexts is needed. Informed by the preparatory qualitative research, this may include a range of study types (e.g. interventional and non-interventional studies considered ‘burdensome’) with differing risk levels, research settings (e.g. care homes, secondary care) and populations/conditions (e.g. acute loss of capacity vs long-term impairing condition).

Participant inclusion criteria:

- Family member or friend approached to act as a personal consultee or legal representative on behalf of a participant eligible for the host trial
- Able to read and understand English sufficiently well to comprehend the study information and decision support booklet
- Able to provide consent to participate in the CONSULT SWAT

Participant exclusion criteria:

- Professional approached to act as a nominated consultee or professional legal representative
- Previously participated in the CONSULT SWAT

### Recruitment

SWAT participants will be identified in accordance with the host trial recruitment processes and their arrangements for recruiting people who lack capacity to consent. Recruitment processes are likely to differ between host trials and may differ between participating sites. As part of aligning the SWAT with the host trial processes, the procedures for identifying eligible participants for the SWAT will be discussed and agreed in advance for each host trial and participating site. The SWAT will use a proportionate approach to informed consent in accordance with HRA guidance [46]. Brief information about the SWAT will be provided to family members approached to act as a consultee or legal representative through a combined information sheet and questionnaire. The information sheet will include the purpose of the SWAT and data protection arrangements, and that completion and return of the questionnaire indicates consent to participate in the SWAT. SWAT participants can also provide their contact details if they are willing to be contacted to take part in an interview for the feasibility stage or process evaluation. SWAT participants and research staff delivering the DA intervention will be purposive sampled to ensure that a range of perspectives are included in the interviews. SWAT participants and research staff agreeing to take part in an interview will provide consent prior to commencing the interview.

### Intervention and allocation

The intervention consists of the DA (a 12-page A5 colour booklet ‘Making decisions about research for others’) which is provided to family members in addition to standard study information about the host trial. The control is standard study information alone for family members. The DA is intended to be used by the family member at the time they are making a decision about whether the person they represent should participate or not. The development of the DA and its contents are described elsewhere [30]. Briefly, it contains an explanation about why they are being approached, why adults lacking capacity are included in research, and a six-step guide to making a decision. It also includes a values clarification exercise to help them to understand what the advantages and disadvantages might be, and to consider how the person they represent would view them and come to a decision about participating or not. The DA is intended to be generic rather than host trial specific; however, the values clarification exercise and other sections help to identify features that are relevant to that particular decision context. The family member is encouraged to identify any areas they feel that they need more information in order to make a decision and to write down any questions they may have in the spaces provided. Depending on the method of communicating with consultees and legal representatives used in the host trial, the DA may be posted out to the participant to be read and completed remotely, or they may be provided with it (in person or remotely) ahead of a consultation with the recruiter (in person or via telephone or video conference) to discuss the participation of the person they represent in the host trial.

**Fig. 2:**
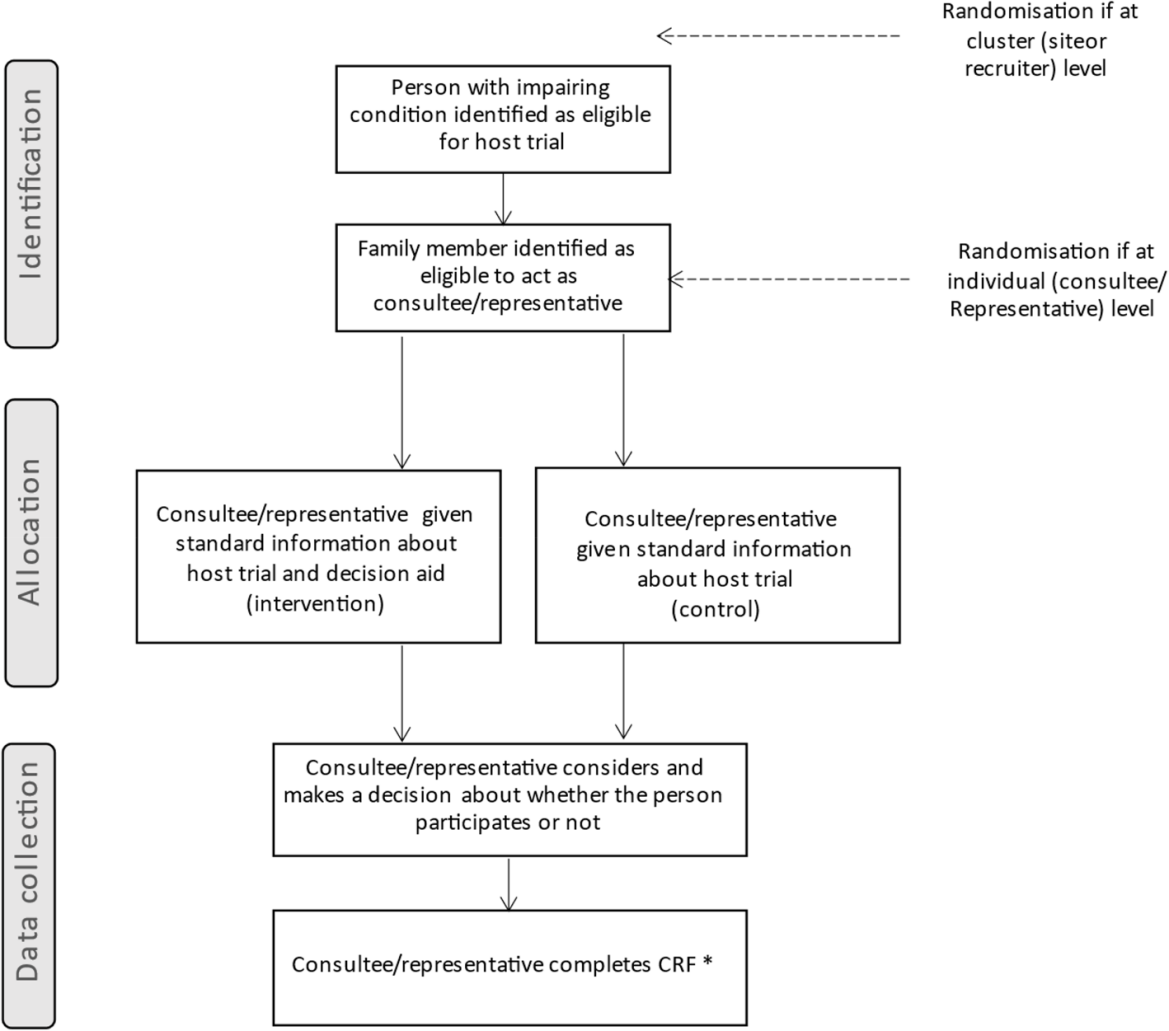
CONSULT SWAT schema. * CRF, Case Report Form. Return of CRF indicates consent to participate

The level of randomisation to either the intervention or control arm will be dependent upon the host trial design, e.g. whether cluster or individually randomised, number of sites and recruiters (see Fig. 2 for the trial schema). Randomisation will preferably be at an individual level (family member) as the intervention is highly amenable to randomisation at that level. But cluster randomisation (recruiter or site) may be required where the host trial itself is cluster randomised, where individual randomisation might cause disruption to the host trial, or where cluster randomisation is the most feasible option for the host trial. Detailed field notes will be maintained to facilitate decisions about randomisation with host trials and to ensure that the randomisation and decision-making process is reported in full. Randomisation will occur in a 1:1 ratio to either intervention or standard information arm. The allocation sequence/algorithm will be generated centrally by the Centre for Trials Research (CTR) who are co-ordinating the CONSULT study. As the level of randomisation will vary depending on the nature of the trial and the feasibility of individual randomisation it may not be possible to maintain blinding. However, SWAT participants will be blinded to allocation (they will not be informed that the study involves randomisation to a DA or control), and site staff involved in data collection for the SWAT will be blinded where possible. This follows SWAT guidance that SWAT participants being aware that different recruitment methods are being tested may impact on their behaviour, thereby confounding the evaluation [31] and is in line with previous SWATs, e.g. [44, 47].

### Outcomes

The primary outcome is the quality of proxy consent decision as measured by the Combined Scale for Proxy Informed Consent Decisions (CONCORD) scale, which is a novel outcome measurement instrument [48]. The CONCORD scale was developed following a consensus study which identified the core outcomes that should be measured when evaluating interventions to enhance proxy decisions and a scoping review which found a lack of appropriate outcome measurement instruments [49]. It has previously undergone feasibility and acceptability testing in a hypothetical decision-making situation [48] and will be concurrently validated during the SWAT. CONCORD scores will be recorded both for consultees and legal representatives who do provide agreement to participation on the person’s behalf and those who decline participation. The timing of outcome measure completion will be aligned with the host trial processes but it is expected to be completed in a relatively short timeframe following the decision.

Secondary outcomes include selected CONCORD subscales of values clarity and preparedness, and where possible the proportion of consultees and legal representatives who provide agreement to participate on the person’s behalf and the proportion who decline participation and subsequent retention in the host trials.

### Data collection

Following receiving the intervention (or standard information alone), SWAT participants will be asked to complete a questionnaire which includes questions relating to demographic data, use and views about the information received, decision outcome (agreeing to participation on the person’s behalf or declining) and the CONCORD scale. The questionnaire will be available in hard copy and online format depending on the host trial processes and will include an option to indicate if the participant is willing to be contacted about taking part in an interview. Where feasible, linked pseudonymised data will be collected on the recruitment and retention of the host trial participant. However, there are likely to be challenges around collecting and sharing this information which we have reported elsewhere [37]. A web-based system (Qualtrics) will be used to enter data online, either directly or from completed paper questionnaires. Interviews will also be conducted remotely with SWAT participants and research staff in both the feasibility stage and process evaluation. Interviews will be semi-structured using topic guides that have been informed by previous qualitative research [23]. Interviews will be audio-recorded and transcribed verbatim and pseudonymised prior to analysis. Data obtained during the interviews will be kept confidential. Voice data will be deleted at the end of the study and anonymised transcripts will be archived in line with Cardiff University policy. Quotes from the interviews may be used in the presentation of results but participants from either the SWAT or host trials will not be identifiable. Anonymised data may be shared with other researchers following the Centre for Trials Research processes for data sharing to ensure confidentiality, regulatory and ethical approvals.

Additional resource use data will be collected and assessed in each host trial using a method that corresponds with the host trial processes for approaching and informing potential consultees and legal representatives. For example, where recruitment is conducted face-to-face or during a consultation, the time required for the consultation and discussion will be recorded where possible.

All quantitative and qualitative data will be stored securely and in compliance with the Data Protection Act 2018 and with the Centre for Trials Research Data Protection and Participant Confidentiality Standard Operating Procedures (SOPs). A Data Management Plan will be in place detailing the management of data for the study.

### Analysis

During the initial feasibility stage, acceptability and feasibility will be assessed through recruitment rates to the SWAT via recruitment logs and CRF return, evaluating completeness of data items, and qualitatively through interviews with family members and research staff (recruiters and members of the trial co-ordination team) including uptake of the invitation to take part in an interview. Provided no changes are required that materially changes the SWAT components, data from the feasibility stage will be subsequently included in the meta-analysis.

In the main SWAT, statistical analysis will be performed using statistical software (Stata). Descriptive statistics will report participant demographic data across the two arms of the SWAT. A two-stage meta-analysis strategy will be used to analyse each individual SWAT taking into account whether it was individually randomised, or cluster randomised to generate trial-level summary statistics, with the results from each individual SWAT then combined across trials. Primary analysis will be on an intention-to-treat basis based on those who return questionnaires. Sensitivity analyses will be undertaken to assess the robustness of the results to non-response to questionnaires, and modelling undertaken if there is a differential response rate between those who receive the decision aid and those who do not.

Where additional data has been collected on recruitment and retention of host trial participants, subgroup analyses will investigate differences between decision outcomes, i.e. whether or not the consultee/legal representative’s decision was that the person should participate in the host trial. Further subgroup analyses and sensitivity analyses may investigate differences between host trial results based on factors such as underlying recruitment rates of the host trials, and timing to outcome measure completion.

Qualitative analyses will be supported by data analysis software (NVivo). At the feasibility stage, interview data will be analysed thematically to provide an understanding of participants’ views about the acceptability of the SWAT, intervention, and outcomes. During the process evaluation, qualitative data from the interviews will be analysed using framework analysis and integrated with the quantitative data to contribute to the assessment of fidelity and other implementation factors. Attention will also be paid to any adaptations that were made in different contexts in order to implement the intervention (which may also undermine intervention fidelity), any contamination between arms or unblinding events, and any changes over time. A key focus of the analysis will be to further develop and test the logic model underlying the decision support intervention to examine the likely mechanisms of action, and the active components.

The contextual factors that may be associated with variation in outcomes will be explored in order to analyse how implementation may vary from one context (host trial, population, setting etc) to another, and whether the intervention has different effects in different contexts even if its implementation does not vary [34]. Contextual moderators will be analysed through detailed description of the nature of the host trial, trial population and consultee/legal representative population, setting, and trial processes that are recorded by the research team through discussion with the host trial team. Qualitative interview data will also be analysed to explore the use and implementation of the decision aid in a range of different contexts.

As part of the concurrent validity testing of the CONCORD scale, factor analysis will be conducted to identify items that most clearly represent the content domain of the underlying construct and any redundancy through testing the internal consistency. Supported by qualitative data from the feasibility stage and process evaluation, this will enable further exploration of existing hypotheses about relationships between constructs and items, and ongoing revision of the underlying theory and construct validation.

The cost-consequence analysis will analyse quantitative resource use data such as the time required for training on the intervention, costs associated with delivering the intervention (e.g. printing, postage), and time required to deliver the intervention, in addition to the usual costs associated with delivering standard trial information. The costs will then be tabulated from the NHS and societal perspectives and presented alongside the CONCORD measure and relevant contextual information using a descriptive table [35].

## Discussion

Given the widely reported challenges of research involving adults with impaired capacity to consent, even moderate effects from the DA on supporting consultees and legal representatives and enabling them to make more informed decisions could be valuable. Through the embedded process and economic evaluation, the SWAT also provides an opportunity to explore implementation factors for the DA, which is the first intervention to enhance the recruitment of adults lacking capacity that is intended for family members. This will generate useful insights for the development and implementation of future interventions for non-emergency trials involving this population. There is also the potential for spill over effects on researchers’ knowledge and confidence in recruiting adults lacking capacity. It may also have a positive effect on attitudes towards research involving adults lacking capacity across the wider research ecosystem. As this is the first SWAT in this population, the protocol provides a template for other researchers who wish to develop and embed a similar SWAT. It therefore contributes to the evidence base for decision-making in trials involving adults lacking capacity, helping to address their current exclusion.

## Trial status

At the time of submission of this article, participant recruitment to this SWAT had not begun. Recruitment to the SWAT is expected to commence in October 2022, with completion by 2025. The SWAT protocol is v1.0 (22.02.2022). Cardiff University is the sponsor for the SWAT. Any changes to the protocol will be communicated to host trials and sites as appropriate.

The findings will be shared with researchers designing and conducting trials involving adults lacking capacity and research delivery teams, as well as trials methodologists with an interest in SWATs. Summaries will be developed in conjunction with the lay advisory group who support the project and provided to family members who participate in interviews, host trial teams, and shared via the CONSULT study website. The findings will be published in peer-reviewed publications and a range of national and international meetings and conferences.

## Data Availability

The dataset that will be generated in this study will be available through submission of a data request to the Centre for Trials Research at https://www.cardiff.ac.uk/centre-for-trials-research/about-us/data-requests.

## Abbreviations

CCA: Cost consequence analysis
CTR: Centre for Trials Research, Cardiff University
CONCORD: Combined Scale for Proxy Informed Consent Decisions
DA: Decision aid
MRC: Medical Research Council
NIHR: National Institute for Health and Care Research
SWAT: Study Within a Trial
RCT: Randomised controlled trial
